# Machine Learning Applications in Suicide Risk Prediction: Data Sources, Modeling Techniques, Ethical Considerations

**DOI:** 10.1192/j.eurpsy.2026.10680

**Published:** 2026-07-31

**Authors:** I. C. Mândraș, A. L. Comșa

**Affiliations:** 1 Psychology and Educational Sciences, Babes-Bolyai University; 2 County Clinical Emergency Hospital, Cluj-Napoca , Romania

## Abstract

**Introduction:**

Psychiatric advancements position machine learning (ML) as a promising tool for suicide risk prediction (SRP). By integrating diverse data streams, ranging from medical records to digital traces and sensor outputs, ML can outperform traditional evaluations that rely on self-report and clinician judgment. Early studies report encouraging accuracy, though questions about generalizability, interpretability, and ethics persist.

**Objectives:**

This review examined how artificial intelligence methods have been applied to SRP, focusing on data sources, modeling strategies, and reported ethical challenges. Our aim was to identify current limitations and outline future research priorities.

**Methods:**

We conducted a systematic search of PubMed using a combination of terms, such as *“machine learning”*, *”deep learning”*, *“risk prediction”*, *“computational model*”*, and “NLP”,* combined with “suicid*”, *“suicide risk”*, *“suicidal behavior”.* The search yielded over 100 results, out of which we selected 35, published between 2020 and 2025. 11 met inclusion criteria for relevance. Both primary research studies and review articles were included.

**Results:**

Data sources

Three data types were used. **Electronic health records(EHRs)** combined structured fields and clinician notes; NLP enhanced detection but faced portability and false positives issues. **Survey and administrative data** offered broad population coverage, improving predictive power, yet coding inconsistencies and demographic gaps limit reliability. **Social media and digital footprints** provided real-time signals, with certain models reaching ~70% accuracy, but they raised ethical concerns. Multi-source integration shows promise but requires careful methodological and ethical oversight.

Modeling Techniques

Data were carefully preprocessed, in order to correct errors, standardize formats, and protect privacy. Non-linear algorithms and neural networks were combined in ensemble methods to capture complex patterns, improve accuracy, and mitigate subgroups bias. Ensembles provided the most precise risk estimates.

Ethical, Legal, and Social Implications

ML for suicide risk involves sensitive clinical, behavioral, and digital data; de-identification, consent, and secure data handling are essential. **Bias and fairness** issues arise from underrepresented subgroups, dataset imbalance, and feature selection; mitigation includes auditing, reweighting, and data diversification. **Transparency and explainability** are vital for trust and clinical adoption; interpretable models, post hoc methods, and clear documentation help clinicians and patients understand predictions.

**Conclusions:**

ML offers a promising path for individualized suicide risk prediction, but ethical deployment requires attention to interpretability and reliability. Integrating diverse data streams and adaptive modeling can support targeted interventions, advancing suicide prevention while maintaining clinical trust.

**Disclosure of Interest:**

None Declared

